# Incorporating human dynamic populations in models of infectious disease transmission: a systematic review

**DOI:** 10.1186/s12879-022-07842-0

**Published:** 2022-11-18

**Authors:** Signe Møgelmose, Karel Neels, Niel Hens

**Affiliations:** 1 Data Science Institute, Interuniversity Institute of Biostatistics and Statistical Bioinformatics, Hasselt University, Hasselt, Belgium; 2 Centre for Population, Family and Health, University of Antwerp, Antwerp, Belgium; 3 Centre for Health Economic Research and Modelling Infectious Diseases, Vaccine & Infectious Disease Institute, University of Antwerp, Antwerp, Belgium

**Keywords:** Demography, Population dynamics, Demographic change, Infectious diseases, Transmission, Mathematical epidemiology

## Abstract

**Background:**

An increasing number of infectious disease models consider demographic change in the host population, but the demographic methods and assumptions vary considerably. We carry out a systematic review of the methods and assumptions used to incorporate dynamic populations in infectious disease models.

**Methods:**

We systematically searched PubMed and Web of Science for articles on infectious disease transmission in dynamic host populations. We screened the articles and extracted data in accordance with the guidelines of the Preferred Reporting Items for Systematic Reviews and Meta-Analyses (PRISMA).

**Results:**

We identified 46 articles containing 53 infectious disease models with dynamic populations. Population dynamics were modelled explicitly in 71% of the disease transmission models using cohort-component-based models (CCBMs) or individual-based models (IBMs), while 29% used population prospects as an external input. Fertility and mortality were in most cases age- or age-sex-specific, but several models used crude fertility rates (40%). Households were incorporated in 15% of the models, which were IBMs except for one model using external population prospects. Finally, 17% of the infectious disease models included demographic sensitivity analyses.

**Conclusions:**

We find that most studies model fertility, mortality and migration explicitly. Moreover, population-level modelling was more common than IBMs. Demographic characteristics beyond age and sex are cumbersome to implement in population-level models and were for that reason only incorporated in IBMs. Several IBMs included households and networks, but the granularity of the underlying demographic processes was often similar to that of CCBMs. We describe the implications of the most common assumptions and discuss possible extensions.

**Supplementary Information:**

The online version contains supplementary material available at 10.1186/s12879-022-07842-0.

## Background

In response to infectious disease threats, mathematical and computational models have proven to be invaluable tools in understanding the spread of infectious diseases in human populations and in quantifying possible disease control strategies as well as evaluating public health interventions, particularly in situations where a controlled trial is ethically or practically unfeasible [1].

The host population studied in an infectious disease model is typically assigned demographic characteristics to account for heterogeneity that may influence the spread of an infection. Population age structure, for example, is commonly included as epidemiological parameters often vary by age, such as the proportion susceptible to immunising infections, which typically decreases with age. Furthermore, contact patterns relevant for the spread of close-contact infections are highly assortative with age, which may affect the exposure to infection. Susceptibility to infection may also vary across ages, as well as the risks associated with an infection [2–4]. Other demographic characteristics and subgroups (e.g. sex, households, schools and spatial structures) may also play an important role in the transmission process of an infectious disease [5–7]. This often implies that the burden of an infectious disease in a population may also be influenced by the relative size of those demographic groups, also referred to as the population composition.

The composition of a population tends to change over time as a result of changes in the underlying demographic processes, which include ageing, births, deaths and population movements. Nevertheless, demographic change is a slow process and is often not incorporated in models of infectious disease transmission, since the time period under consideration tends to be short. Moreover, it is often useful to disregard demographic change when focusing on how epidemiological factors alone influence different outcomes [8]. For some infections, settings and research questions, however, the realism of the population composition and how it evolves play an important role. This often applies to analyses of disease transmission dynamics and public health interventions over a longer time frame, where demographic changes are to be expected. Fertility declines, for example, have in some cases been linked to increases in the average age at infection of diseases traditionally considered to be childhood diseases [8–12]. This may affect the disease burden of infections associated with increased morbidity and mortality in certain age groups or during age-related events such as pregnancy [2]. The burden of infections with a higher incidence and severity among the elderly is also expected to increase as a population undergoes ageing, as has been seen with herpes zoster [13, 14]. Such relationships can only be investigated by allowing for demographic change in the host population.

Demographic change can be introduced in models of infectious disease transmission in various ways. The population can be subjected to constant fertility and mortality rates for an extended period of time, where demographic change will result from the gradual convergence of the population to the implied stable population with a constant relative age distribution and a fixed growth rate [15]. In many cases, this provides a useful approach for investigating disease transmission dynamics in a population with a changing composition induced by preceding trends in fertility, mortality and migration. However, as the time period expands, the assumption of constant demographic rates becomes implausible. Thus, for the evaluation of long-term effects, it may be important to consider demographic change in the host population by explicitly considering and including dynamic demographic processes. We will refer to this as a dynamic population which is the main focus of this paper.

Dynamic populations are incorporated in an increasing number of mathematical and computational models for infectious disease transmission. These models have shown an important impact of demographic change on the long-term dynamics of infectious diseases, as well as for the effectiveness of immunization programmes. For example, long-term demographic changes have been found to have a considerable effect on the epidemiology of varicella and herpes zoster, implying that the demographic assumptions have an impact on the predicted burden of disease [14, 16].

The demographic methods used to incorporate a dynamic host population in models of infectious disease transmission vary considerably. The methods range from adjusting the age distribution over time according to population projections to complex models with dynamic demographic processes and subgroups such as households [e.g. 9, 14]. This includes population-level models and individual-based models (IBMs). We use the term ‘IBM’ to refer to all models at the individual level, including microsimulations and agent-based models [17]. IBMs are increasingly used to model disease transmission, however, it is unclear whether their flexibility also enhances the level of detail incorporated in the demographic modelling. Finally, various demographic assumptions are applied in models of disease transmission, such as no migration, but the implications thereof are not always explained.

With this systematic review, we provide an overview of the methods and techniques used to model dynamic population structures in the context of infectious disease modelling, which to our knowledge has not previously been attempted. We discuss the advantages and limitations of various modelling techniques in order to improve the understanding needed to evaluate their suitability in a given study. Moreover, we discuss the potential implications certain demographic methods and assumptions have for the population composition and potentially for the epidemiological outcomes. As previously mentioned, dynamic host populations are in many cases not incorporated in models of infectious disease transmission and typically for good reasons. Thus, the aim is to identify the smaller group of infectious disease models where dynamic population structures have been a major point of attention. To obtain this, while taking the feasibility of the search into account, we limit our review to infectious disease models with a focus on demographic change. We differentiate in terms of the method that is used to model the host population, the different demographic processes, as well as the data and techniques used to model each demographic process.

## Methods

We carried out a systematic review in accordance with the guidelines of the Preferred Reporting Items for Systematic Reviews and Meta-Analyses [18]. The methods and procedures are described in a protocol (Additional file 1).

### Search

We searched PubMed and Web of Science Core Collection for articles published up to August 25th 2020 without language or time restrictions using the following search string in titles and abstracts:

(demography OR “demographic transition” OR “demographic change*” OR “population change*” OR "household structure*" OR "household composition*" OR "population ageing" OR "population aging" OR "aging population" OR "ageing population") AND (infect* OR vaccin* OR epidemic* OR communicable) AND (model* OR simulat*) NOT (animal* OR plant*).

The asterisk in some search terms represents any group of characters, including no character (e.g. infect*: infected, infection, infectious etc.). The search string includes terms related to demography since we mainly expect dynamic host populations to be incorporated in papers with a focus on demographic change. Broader search terms (e.g. demograph*) and the search of full-text and supplementary material would provide a more thorough search but would result in an unfeasible amount of hits. A detailed overview of the result of each search term and the overall search strategy is shown in Additional file 1: Table S1. The results of the search strategy were managed in Endnote X9.

### Eligibility criteria

The eligibility criteria were defined by two researchers (SM and NH) prior to screening. We included research papers on mathematical and computational models for infectious disease transmission in a human population. The host population should at least be divided into five age groups. Moreover, the population should result from a model including at least fertility and (all-cause) mortality as dynamic processes. No requirements are made for disease-specific mortality, if included in the model.

**Fig. 1:**
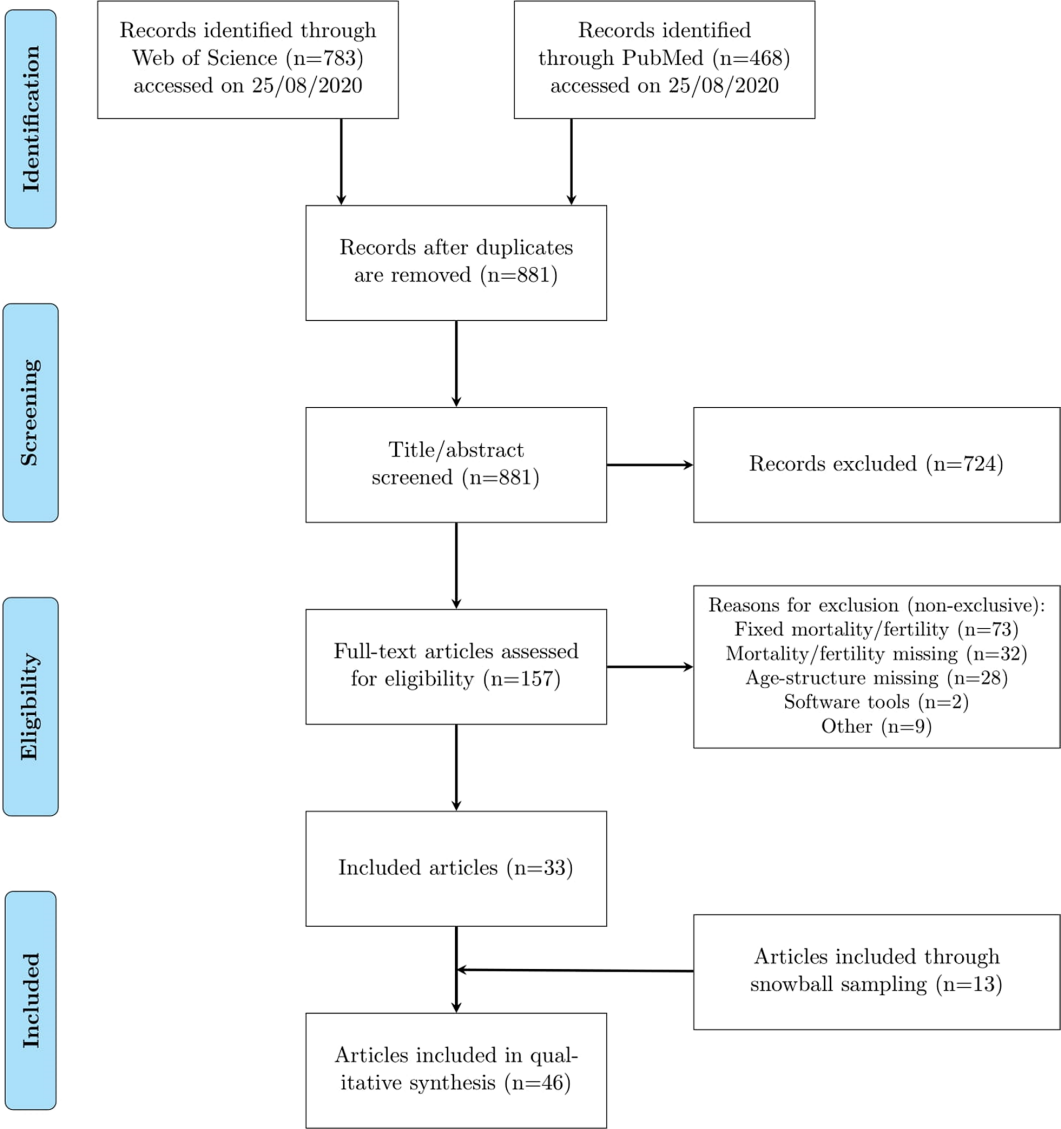
PRISMA flow diagram of the article selection process

The demographic model can be included explicitly or population structures from another source can be used as input to the disease transmission model, as long as this population is the result of a demographic model explicitly considering dynamic trends for fertility and mortality. This implies that models assuming constant fertility or mortality rates throughout the entire study period are excluded. However, models with constant rates in a limited part of the study period are still included. Articles only describing the technicalities behind a method or a software tool without applying it to any population are also excluded. Finally, models limited to high-risk groups (MSM community, injecting drug users etc.) are excluded. The screening and selection processes are presented in Fig. 1. Titles, abstracts and full-texts were screened by a single reviewer (SM) and discussed with the last author (NH) in case of doubt. We also identified articles by screening reference lists of the included articles.

### Data extraction and analysis

For all eligible articles, we retrieved and classified information as follows: (1) Setting and population characteristics: country/region, population, demographic characteristics and time horizon; (2) Model specifications and data: model type, demographic processes considered and source of demographic data; (3) Modelling of demographic processes: starting population, fertility, mortality, migration, household networks and sensitivity analysis of demographic assumptions; (4) Specifications of disease transmission model and analyses: disease, vaccination, social mixing and cost-effectiveness analyses. Models from the same article were included if they were eligible and differed from each other in setting, model specifications or demographic processes. To make it clear that models originated from the same article, letters were added to the article number in figures and tables. Different articles applying the same modelling tool have all been included and the article numbers are followed by an abbreviation for the modelling tool when visualising demographic processes.

## Results

We identified 881 articles (after removing duplicates) by searching the databases PubMed and Web of Science Core Collection with the search string mentioned under Methods. Based on the defined eligibility criteria, we screened titles and abstracts and excluded 724 articles. For the remaining articles, a full-text analysis was carried out in case fulfilment of the eligibility criteria was uncertain. Most articles excluded at this stage were assuming constant fertility and/or mortality rates. We identified 13 articles through snowball sampling. Finally, 46 articles, containing 53 different models, were included in the qualitative analysis. The data retrieved from each article can be found in Additional file 1: Tables S2–S9. In this section, the term ‘study’ is used to refer to all models within one article.

### Setting and time period

**Fig. 2:**
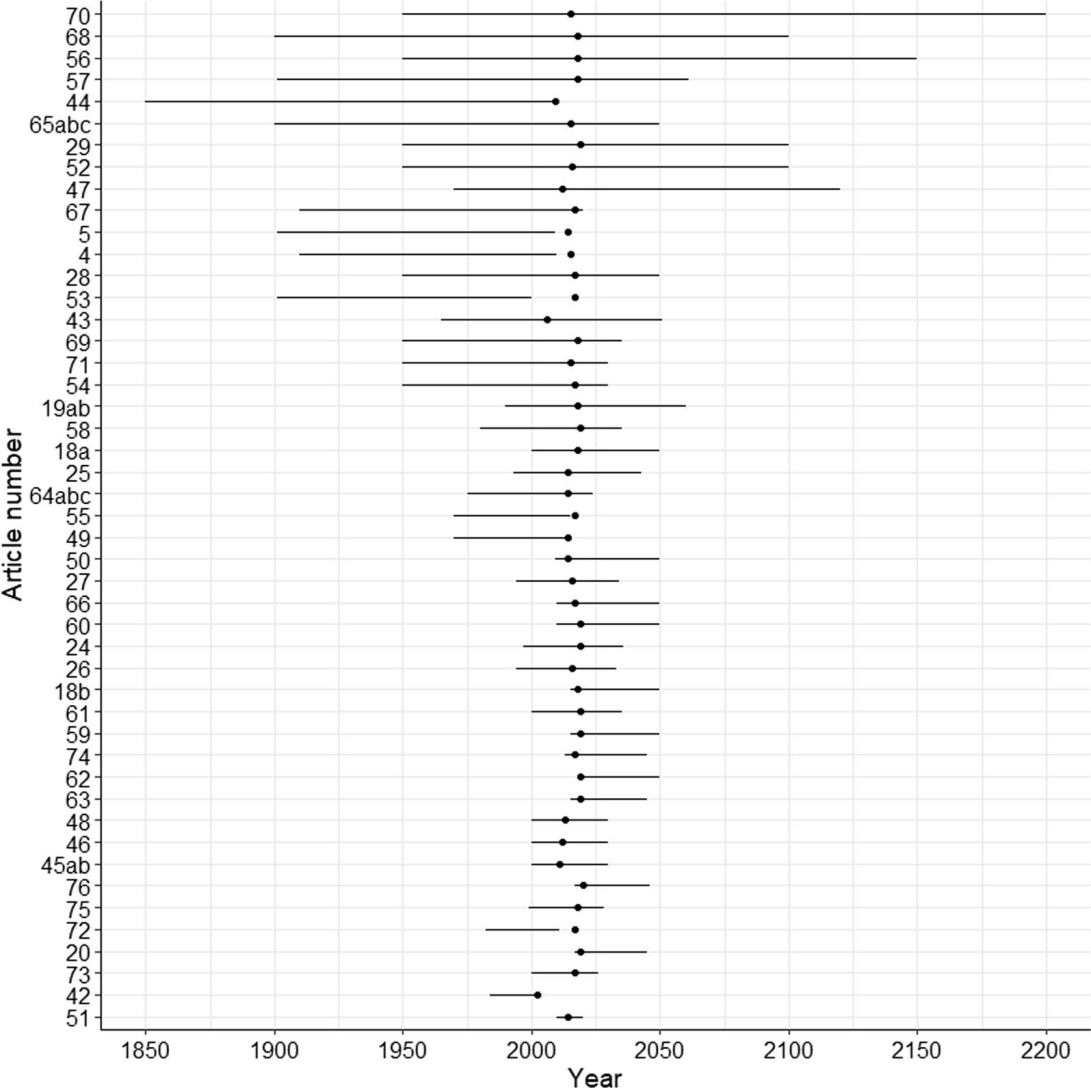
Modelling period by article number in the reference list (points indicate years of publication)

In the included studies, populations were modelled for countries, regions and cities in Europe (20), Asia (16), Africa (14), North America (12), Oceania (9) and South America (6). Twelve studies covered multiple populations. In most of the studies, past as well as future time periods were modelled, six studies only covered a historical period while five studies only looked at projections of the future. The length of the modelling period varied between 10 and 250 years as seen in Fig. 2.

### Model type

We divide the demographic models in the included studies into three types: (i) disease models that use demographic population prospects as an external input (EPMs: external population models), (ii) cohort-component-based models that use cohort-component projections to model demographic change (CCBMs) and (iii) individual-based models that model demographic events at the level of individual life courses (IBMs).

First, EPMs draw the annual population composition from an external source and use it as an input for the disease transmission model instead of modelling the demographic processes explicitly. Given assumptions for the different components of demographic change, statistical agencies often generate projection sets that provide annual information on population composition, typically by age and sex. In this approach, the population composition is allowed to vary over time, but population dynamics cannot be attributed to changes in fertility, mortality or migration separately, because only the resulting population composition is used.

Second, in CCBMs, the population is divided into subgroups to which group-specific rates for fertility and mortality are applied in each projection step to work out population change over time. In most cases, CCBMs do not consider household or family dynamics. Depending on the assumptions made, emigrants and immigrants are subtracted and added, respectively, by age and sex [15]. As is the case for the EPMs discussed earlier, CCBMs are typically integrated into a compartmental disease transmission model by adding the demographic sub-groups (e.g. age groups) to each compartment. As a result, both disease transmission and population dynamics are modelled at the population level.

Third, in IBMs, the unit of analysis is the individual. In IBMs, all individuals are assigned a set of attributes (e.g. age, sex, marital status) and are in every time interval subject to covariate-specific risks of demographic events, such as union formation or dissolution, fertility, mortality and migration [19]. However, the number of covariates included in each demographic process varies substantially between the included IBM studies. Given the predicted probabilities, a random number generator is used to determine whether an individual experiences the event and the individual’s attributes are updated accordingly [20]. This makes it possible to track the life course of each individual. In order to simulate disease spread in a demographic IBM, a disease state is added to the individual attributes. Moreover, interactions between individuals as well as subgroups (e.g. households, schools) and network structures (e.g. mobility networks) can be included when relevant for disease transmission [1], which was done in several studies.

**Fig. 3:**
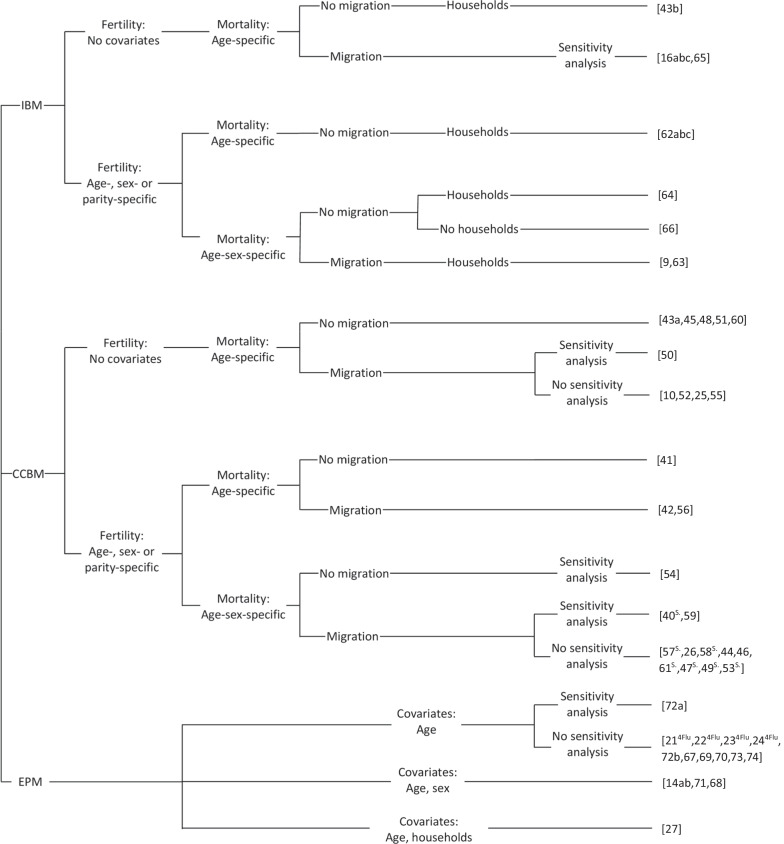
Branching diagram of model type, demographic processes and covariates with article number in brackets (S.: Spectrum software, 4Flu: 4Flu model)

In 14 studies, existing population prospects were used as external input in the disease model (see Fig. 3). CCBMs and IBMs were applied in 24 and 7 studies, respectively, while one study applied both approaches. This implies that the majority of the included studies use population-level models, but fertility, mortality, and in some cases also migration, were in most cases modelled explicitly.

### Starting population

Most studies based the starting population on the observed population composition in the first year of the modelling period or on a population sample. In 18 studies, however, the starting population was generated by simulating demographic events and disease transmission in an initial population using a set of demographic rates for a defined period of time (see Additional file 1: Tables S2–S3 for more detail). In this way, an epidemiological equilibrium can be obtained in the starting population, while respecting any specified demographic constraints (e.g. demographic generation intervals, birth intervals). In most of these studies, the fertility and mortality rates remained constant in the initialisation period, which eventually leads to a stable population. The relative age distribution of a stable population is not influenced by the initial age distribution, but is entirely determined by the fertility and mortality rates assumed [15]. Consequently, the age composition in the initial non-stable population and the resulting stable starting population may differ considerably. Some studies compensated for this by adjusting the demographic rates used to generate the starting population. Household membership was included in the starting populations of the eight models incorporating households. Individual-level data on household position and composition were lacking in most studies and marginal distributions of household size and age compositions were applied instead. Different algorithms and constraints were applied to obtain somewhat realistic age differences between household members in the starting population. In several studies, household members were assigned different positions (e.g. adult in a union, single adult, child) based on data or defined rules.

### Demographic processes

The 32 studies using IBMs and CCBMs included at least dynamic trends for fertility and mortality. In most of these studies, covariates were included in the fertility and mortality processes (e.g. age and sex), as seen in Fig. 3 and further described below. Migration was included in 22 studies and households were only incorporated in eight models. Finally, demographic sensitivity analyses were performed in nine models. In some EPMs [21–24], it was assumed that the number of births in a given year equals the size of the youngest age group, while a decrease or increase over time in all other age groups was ascribed to mortality and immigration, respectively. These details are not included in Fig. 3 because the changing size of an age group cannot be ascribed to one demographic process alone. The external population prospects typically result from a set of assumptions for fertility, mortality and migration, but since these are not modelled explicitly in the infectious disease model, EPMs are not considered in the further discussion of the demographic processes. The subgroups by which the population in an EPM is decomposed are described in Fig. 3 instead.

#### Fertility

In order to model past fertility patterns, the large majority of models used observed fertility rates, probabilities or birth numbers, as shown in Additional file 1: Fig. S1 and Table S4, which in most cases were obtained from official statistical agencies. In case annual estimates of vital statistics were not available, interpolation, averages over multiple years or step-wise functions were used. Only three models applied a scenario-based approach with an assumed trend.

A larger variety was seen in the methods for projecting future fertility trends. Official fertility projections were applied in 15 models, which were mainly taken from national statistical agencies or the United Nations World Population Prospects. Eight models carried the last observation forward, meaning that future fertility trends were assumed to remain at the last observed level. One study combined the previous two approaches by applying official projections for the years available, and for the remaining projection period, the last projected value was carried forward. Authors developed their own scenarios for future fertility trends in five models, either expressed as a yearly percentage change or as policy scenarios, while extrapolation was applied in one model. Finally, the applied approach was unclear in one model (the author was contacted but does no longer have access to the information).

About the same proportion of IBMs and CCBMs, which amounted to 15 models in total, included no covariates when modelling fertility. In most of these cases, crude birth rates (CBR) were used. Age was included in the fertility process in one model and 16 models included sex as well as age, meaning that age-specific fertility rates (ASFR) were applied to females in their fecund age range (typically 15–49 years of age). Five models, which were all IBMs, took birth parity (number of children ever born to a female) into account, in addition to age and sex, and two of these also considered birth interval by assuming a minimum amount of time between subsequent births for a given female.

#### Mortality

Past (all-cause) mortality patterns were modelled using observed mortality rates or numbers of deaths in most models as shown in Additional file 1: Fig. S2 and Table S5. Interpolation, averages over multiple years or step-wise functions were applied in cases where yearly estimates were not available. Future mortality patterns were modelled using official projections in 16 models and 10 models used the most recent observation for the whole projection period. Other methods, including extrapolation and the scenario-based approach, were applied in a smaller number of models. Mortality was age-dependent in 21 models and age-sex-dependent in 16 models. Furthermore, several models included disease-related mortality or certain risk factors (not included in Additional file 1: Fig. S2).

#### Migration

Migration was included in 24 models in the form of net migration and mainly obtained from official estimates and projections (see Additional file 1: Table S6). Three of these models assumed that the composition of the migrant population was similar to that of the native population, but it was acknowledged in the studies, that in reality these tend to differ markedly [9, 25, 26]. The age and age-sex distributions of the net-migrant population were considered in 9 and 10 models, respectively. In most of these cases, a lack of data made it necessary to assume that the composition of the net-migrant population remains constant over time. Internal migration was modelled in a broad sense in three models, namely as migration between rural and urban areas and without considering any covariates. Migration was incorporated in the majority of CCBMs and in about half of the IBMs.

#### Households

Households were incorporated in eight models, of which seven were IBMs and one was an EPM [27] (see Additional file 1: Table S7). The households evolved dynamically over time in all models. In the IBMs, individuals move between households or create new households (e.g. child leaving parental household). In most cases, the probabilities for household transitions were fixed over time and equal across all ages eligible for a given transition. Households were also dynamic in the EPM, however, a new population of individuals was generated and assigned to households at the beginning of each simulation year according to an algorithm using the observed or projected age composition and the last observed age distribution by household size. This implies that changing household structures could not be traced back to individual household transitions.

### Sensitivity analyses

Sensitivity analyses of population projections were included in a small number of models (see Additional file 1: Table S8). Alternative scenarios for the overall age distribution or for fertility, mortality and/or migration separately were applied in seven models, two models quantified the uncertainty of the demographic parameters with confidence and prediction intervals, respectively, while one model compared step-wise functions with interpolation between five-year estimates.

## Discussion

We identified 46 studies, which contained 53 models for infectious disease transmission in populations with dynamic demographic processes. The dynamic population models in the included studies varied in the methodology, the demographic characteristics and processes included in the model and overall complexity. Population-level models, EPMs and CCMBs, were most common, while individual-based models were least common. This was to be expected, as the use of IBMs in infectious disease modelling is relatively new, but has been expanding in recent decades [17]. Moreover, fertility, mortality and migration were modelled explicitly in most models (CCBMs and IBMs), while EPMs using population prospects from a statistical agency as external input made up a smaller share of the models.

One demographic model cannot generally be considered superior to another because its suitability must be evaluated jointly with the infectious disease model and the aim of the given study. However, the demographic methods and assumptions serve different purposes and are associated with different limitations and possibilities for extensions.

In EPMs, the population composition changes over time as individuals enter and leave the population, but the demographic change cannot be traced back to fertility, mortality and migration separately, since these processes are not modelled explicitly in the infectious disease model. The underlying demographic assumptions are often available from the statistical agencies developing the population prospects. The population heterogeneity in EPMs is determined by the level of detail provided in the population prospects, which often is limited to the age or age-sex composition. In infectious disease modelling, however, demographic variables additional to age and sex are often not required. Thus, EMPs provide a straightforward and relatively simple implementation of a dynamic host population in cases where it is not of interest to consider fertility, mortality and migration separately. However, it is worthwhile to explicitly state and discuss the demographic methods and assumptions underlying the population projection, even when they are not modelled explicitly, as different demographic assumptions may give rise to quite different population dynamics, which in turn may affect the epidemiological outcomes.

In CCBMs, the processes that generate changes in the population composition are explicitly incorporated as assumptions or sub-models. Thus, a strength of CCBM is the possibility to assess variation in epidemiological outcomes given alternative scenarios for each of the demographic components. This is particularly relevant when considering time periods far into the future. Moreover, characteristics beyond age and sex can be included in a CCBM, but the system of demographic subgroups and disease state compartments becomes quickly very complex. Consequently, none of the included CCBMs incorporated demographic subgroups beyond age and sex.

The highest degree of flexibility is provided by IBMs, which make it possible to include more heterogeneity in both the population and the disease transmission process. The majority of the IBMs (60%) included households as well as other demographic subgroups (e.g. schools, workplaces). Demographic subgroups and networks are important for the transmission process of many infectious diseases. Households especially play a central role due to the higher frequency and intimacy of social contacts among people living together [6, 28]. Individual-based modelling may also make it easier to track the life course and/or health trajectory of individuals. Thus, past events can be taken into account, when determining the probability of future events. Five IBMs, for example, included birth intervals and/or parity in the fertility process, which would be more difficult to accomplish in a population-level model.

Nevertheless, similar methods were used to model fertility, mortality and migration in the majority of models explicitly incorporating demographic processes (CCBMs and IBMs). In about 40% of these models, the number of births was modelled proportionally to the size of the total population (crude birth rate), disregarding the age structure, and changes therein, of the female population [29]. As a result, the number of births in the dynamic population will only be correct as long as the age-sex composition remains similar to that of the population on which the crude rate was based. Age-specific fertility rates (ASFRs), which are directly standardized for age-sex composition, are preferable in that respect and were used in most of the remaining studies. To the extent that characteristics of household members, parents or siblings (e.g. age, vaccination status) or other kinship-related factors play a role in the disease transmission process, it is necessary to incorporate these characteristics in the fertility process as well. This was seen in the aforementioned IBMs, which included birth interval and parity in the fertility process in order to obtain appropriate generation intervals and household compositions.

The relationship between (all-cause) mortality and age was acknowledged in all IBMs and CCBMs and about half also considered the impact of sex. In most settings, however, mortality in the middle and older ages has been shown to vary by a considerably larger set of factors, including household composition, living arrangement and marital status, especially among males [30]. This could be particularly relevant to take into account when modelling ageing populations, where household structures are increasingly influenced by the developments in the elderly population. However, this would require very detailed data which often is not accessible. Thus, characteristics beyond age and sex were unsurprisingly not considered in the mortality process of any of the included studies.

In addition to fertility and mortality, about two-thirds of the CCBMs and IBMs modelled migration. A typical age pattern is often observed in migration, with a peak in young adulthood and in childhood, because of children joining their parents in migration. In high-income countries, a smaller peak is also observed around the retirement ages [15]. This was incorporated in the majority of the studies modelling migration. Migration patterns are highly complex and typically associated with a high degree of uncertainty [31, 32], which could partly explain why migration was not taken into account in about a third of the models. However, in most countries, migration flows cannot be considered negligible and changes in population size and composition may be biased if migration is ignored [33]. Statistical agencies typically provide estimates of past as well as projected net migration.

Households and other subgroups were incorporated in eight models, which all were IBMs. Such structures are more cumbersome to implement in population-level models, thus individual-level modelling seems to be preferred if demographic subgroups are considered important for the disease transmission process, setting or research question at hand. Despite the flexibility of IBMs, it remains complex to model household structures. Detailed data on household characteristics, in particular historical data, is very limited, which can make it necessary to make strong assumptions regarding household structures. For example, several studies assigned individuals to households according to marginal distributions of household size and composition rather than individual- or household-level data, which often is unavailable.

However, households did evolve dynamically over time in all models, which is important for assessing the impact of demographic change on disease transmission dynamics. Declining household sizes and changing compositions are resulting from population ageing due to decreasing fertility rates and rising life expectancy [34]. Consequently, the number of household contacts decline and the age structure in the household contacts changes [9]. This implies that boosting of immunity through household transmission becomes less likely [35].

Most studies, however, focused on the most common household types and/or positions and left out the rest completely, or gathered them in one category. In many cases this approach may be warranted given that the required level of demographic precision is, in this context, determined by its relevance for disease transmission, the setting and research question. Nevertheless, some household types may be important for the disease spread and burden, even if they represent a relatively small proportion of the population. For example, nursing homes and other special care facilities for the elderly make up a small share of the households in most countries but provide an optimal environment for the spread of many infections [36]. Moreover, the enhanced age and underlying chronic illnesses place this population group at increased risk of many infections [37, 38]. Thus, in some contexts, less common household types can be important to consider if the data is available.

Future demographic trends are associated with a large degree of uncertainty, which is an important aspect in projecting populations [31]. Thus, an assessment of this uncertainty and its impact on epidemiological outcomes by the means of sensitivity analyses is highly relevant. Statistical agencies often provide different scenarios or prediction intervals for future fertility, mortality and migration levels, which can be used for this purpose. A smaller number of included models (10) performed sensitivity analyses by assessing the impact of variation in demographic trends on epidemiological outcomes.

The findings of this systematic review should be considered in light of several limitations. More studies may have been relevant to include but were not captured in the search due to the requirement of a reference to certain demographic terms in the title or abstract. However, the number of hits would be unfeasible to handle if the demographic search terms were omitted and if searching full-text and supplementary materials. Moreover, static and stable host populations are most common in infectious disease modelling and the incorporation of dynamic demographic processes involves a degree of complexity that we would expect most researchers to omit unless the study has a specific focus on the impact of demography or demographic change. Note that, to minimise any potential bias, we also conducted snowball sampling.

## Conclusions

We systematically reviewed the literature on infectious disease modelling with a dynamic host population. We found that population-level modelling (EPMs and CCBMs) was more common than individual-based modelling. EMPs provide a straightforward and relatively simple implementation of a dynamic host population, while CCBMs are a bit more complex but make it possible to consider each demographic process separately and to test different demographic assumptions. Demographic characteristics beyond age and sex were only included in IBMs, including birth interval and parity, as well as households and other demographic subgroups. However, we found that the majority of IBMs modelled fertility, mortality and migration in a similar manner to the CCBMs, namely by the use of crude rates or age-(sex)-specific rates. We recommend avoiding the use of crude rates, if possible, as they disregard the population age structure and changes therein. In addition to fertility and mortality, we recommend including migration in the demographic model, since most countries face substantial migration flows and changes in population size and composition may be biased if migration is ignored. The approach used to model each demographic process implies certain assumptions, and the implications these may have for the population composition should be given careful consideration, and above all be stated clearly. Finally, the inherent uncertainty in demographic trends and their potential impact on epidemiological outcomes is ideally addressed using sensitivity analyses.

## Supplementary Information

**Additional file 1: Protocol, figures and tables.**

## Data Availability

All data generated or analysed during this study are included in this published article and its supplementary information files.

## Abbreviations

IBM: Individual-based model
CCBM: Cohort-component based model
EPM: External population model
CBR: Crude birth rate
ASFR: Age-sex-specific fertility rate
MSM: Men who have sex with men
S: Spectrum software
4Flu: 4Flu model
