# Supplementary material for: Incorporating human dynamic populations in models of infectious disease transmission: a systematic review
Source: BMC Infect Dis. 2022 Nov 18;22:862. doi: 10.1186/s12879-022-07842-0 (PMC9673416; doi:10.1186/s12879-022-07842-0)
Supplement: Supplementary file 1 — Additional file 1: Protocol, figures and tables. [file 12879_2022_7842_MOESM1_ESM.docx]

**Appendix**

**Protocol for the systematic review of incorporating human dynamic populations in models of infectious disease transmission**

# Background

The host population in models of infectious disease transmission is typically based on rather strong assumptions regarding the demographic composition and how it changes over time. Stable populations, meaning that the relative age composition remains constant over time, are often used [4,8]. Such assumptions have proven to be useful to gain epidemiological insights and may be justified for disease outbreaks taking place over shorter time frames during which no considerable demographic change is expected [39]. In reality, however, populations never reach stability as fertility and mortality levels are subject to continuous change. Furthermore, the demographic processes and their changes over time may influence social contact patterns with importance for disease transmission. This is acknowledged in an increasing number of mathematical and computational models for infectious diseases, which incorporate changes in the population structures over time. These models have shown an important impact of demographic change on the dynamics of infectious diseases, as well as for the effectiveness of immunization programmes (see Geard et al. [9], Horn et al. [14], Marziano et al. [27]). Nevertheless, the included population dynamics and demographic modelling approach vary highly from model to model. The methods used to incorporate dynamic population structures in infectious disease models has to our knowledge never been systematically summarised.

# Objectives

The objective of this systematic review is to summarise and discuss the methods that have been used to incorporate dynamic population structures into models for infectious disease transmission. This includes the methods used to model the host population, the different demographic processes considered, as well as the data and techniques used to model each demographic process. The questions to be answered in the systematic review are:

- Which methods are used to model a dynamic host population?
- Which demographic processes are explicitly incorporated in infectious disease models with a dynamic population?
- How are the demographic processes modelled and which data is used?

# Methods

## Eligibility criteria

We will search for publications of mathematical and computational models for infectious disease transmission in a human population that results from a model including at least fertility and mortality as dynamic processes. The demographic model can be included explicitly or a population from another source can be used as input to the disease transmission model as long as this population is the result of a demographic model explicitly considering dynamic trends for fertility and mortality. This implies that models assuming constant fertility or mortality rates throughout the entire study period are excluded. However, models with constant rates in a limited part of the study period are still included. The inclusion and exclusion criteria are listed below.

Inclusion criteria:

- Mathematical and computational models for infectious disease transmission among humans
- A dynamic population (observed or synthetic)
- Fertility and mortality are modelled as dynamic processes
- A population modelled with dynamic fertility and mortality trends by another source

Exclusion criteria:

- Less than five age groups
- Use of stable populations
- Fixed mortality or fertility rates throughout the entire study period
- Population modelled with intervals in time of 10 or more years (e.g. 2020, 2030)
- Models limited to high-risk groups (MSM community, injecting drug users)
- Non-communicable disease
- Technical papers or software tools without any application
- Reviews (unless it is a review of incorporation of demography in infectious disease models)

## Information sources

We will search the electronic databases Web of Science and PubMed from the earliest date of the database to 25.08.2020. Additionally, we will carry out a manual search by screening reference lists of included papers.

## Search strategy

The following search string is used to search in titles and abstracts:

- demography OR “demographic transition” OR “demographic change*” OR “population change*” OR "household structure*" OR "household composition*" OR "population ageing" OR "population aging" OR "aging population" OR "ageing population"
- AND (infect* OR vaccin* OR epidemic* OR communicable)
- AND (model* OR simulat*)
- NOT (animal* OR plant*)

# Study records

## Data management

We will make use of the reference software EndNote X9 to manage the identified publications.

## Selection process

Titles and abstracts will be screened by using the inclusion and exclusion criteria stated above. Articles will be reviewed in full-text in case of doubt.

## Data collection process

The reviewer will extract the data using a standardised form. Papers might be excluded at the data collection stage if it becomes apparent that inclusion criteria are not met or if there is not enough information in the paper to extract the required data.

## Data items

The extracted data will include the following:

- Setting and population characteristics
  - Country/region/city
  - Population
  - Demographic characteristics (age, sex, etc.)
  - Time horizon
- Model specifications and data
  - Model type
  - Demographic processes considered
  - Source of demographic data
- Modelling of demographic processes
  - Starting population
  - Fertility
  - Mortality
  - Migration
  - Households and networks
  - Demographic sensitivity analyses
- Specifications of disease transmission model and analyses
  - Disease(s)
  - Vaccination
  - Social mixing
  - Cost-effectiveness analyses

# Data analysis

The qualitative data analysis will include:

- A flowchart describing included and excluded articles
- Tables presenting the characteristics of the included articles with information regarding
  - Setting and population characteristics
  - Model specifications and data
  - Modelling of demographic processes included in study
  - Components of disease transmission model
- Figures visualising
  - Model types
  - Demographic processes included in model
  - Demographic data

**Figures and tables**

Table S1: Search strategy and hits

| **#** | **Search** | **Web of science: Abstract/title** | **PubMed:  Abstract/title** |
| --- | --- | --- | --- |
| 1 | "demographic transition" | 2.353 | 1.237 |
| 2 | demography | 27.772 | 10.980 |
| 3 | "demographic change*" | 6.641 | 3.117 |
| 4 | "population change*" | 5.310 | 2.137 |
| 5 | "household structure*" | 915 | 344 |
| 6 | "household composition*" | 1.037 | 519 |
| 7 | ("population ageing" OR "population aging") | 4.561 | 2.663 |
| 8 | ("aging population" OR "ageing population") | 11.861 | 10.280 |
| 9 | infect* | 1.899.379 | 1.784.209 |
| 10 | vaccin* | 349.222 | 318.058 |
| 11 | epidemic* | 117.329 | 108.101 |
| 12 | communicable | 12.809 | 18.047 |
| 13 | model* | 9.303.490 | 2.931.098 |
| 14 | simulat* | 3.292.060 | 535.675 |
| 15 | 1 OR 2 OR 3 OR 4 OR 5 OR 6 OR 7 OR 8 | 57.934 | 30.033 |
| 16 | 9 OR 10 OR 11 OR 12 | 2.175.981 | 2.055.072 |
| 17 | 13 OR 14 | 10.933.783 | 3.228.393 |
| 18 | 15 AND 16 AND 17 | 869 | 652 |
| 19 | 18 NOT (animal* OR plant*) | 783 | 468 |


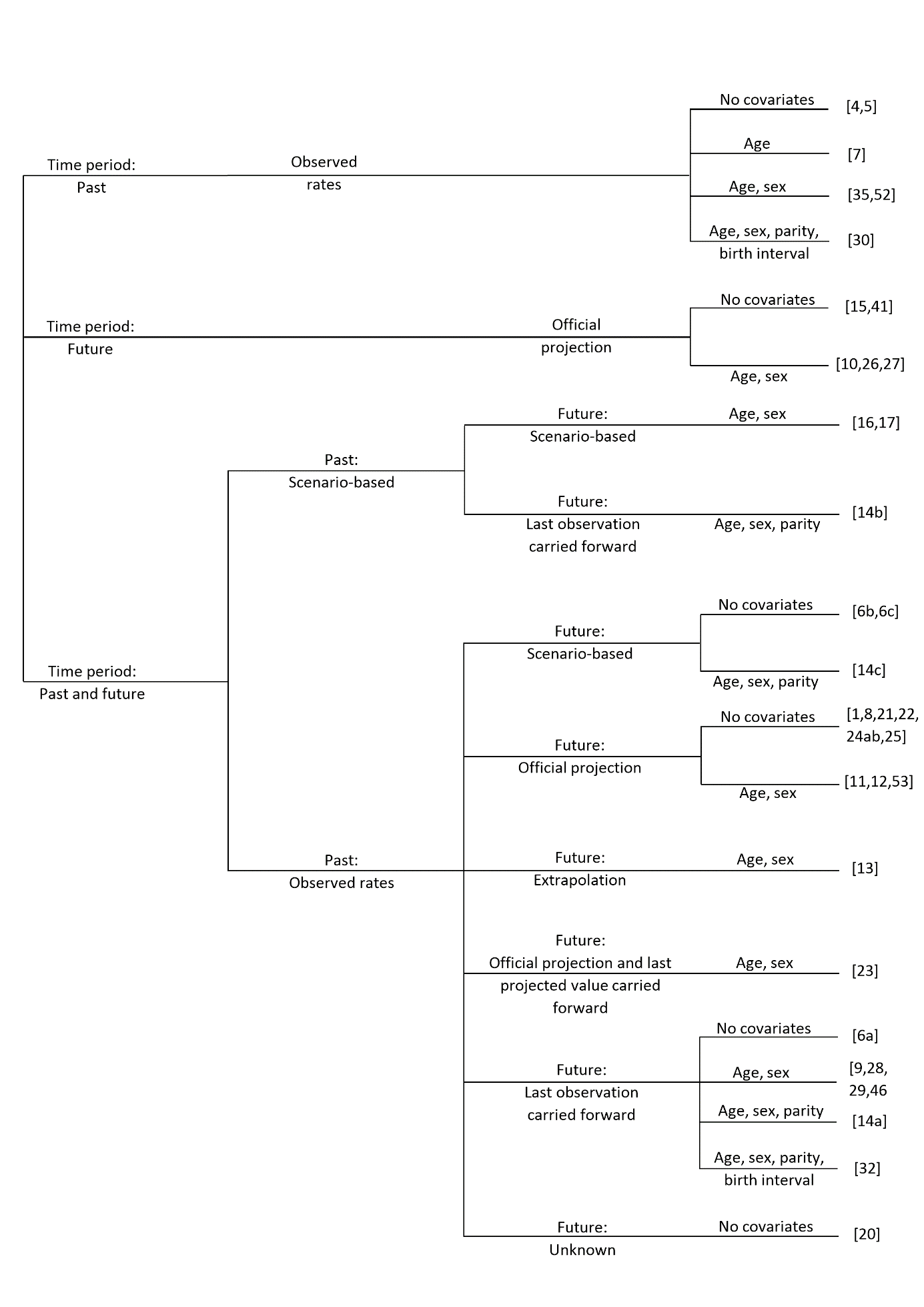


[10,51]

[9]

[45]

[54]

[62a]

[59]

[57^S.^,61^S.^,63]

[42]

[40^S.^,41,56,66]

[26,49^S.^,58^S.^]

[64]

[16a]

[25,43a,43b,50, 52, 55, 65]

[62c]

[16b,16c]

[62b]

[44,46]

[48,60]

[47^S.^,53^S.^]

*Figure S1: Branching diagram of fertility modelling with article number in brackets. (S.: Spectrum software, 4Flu: 4Flu model).*


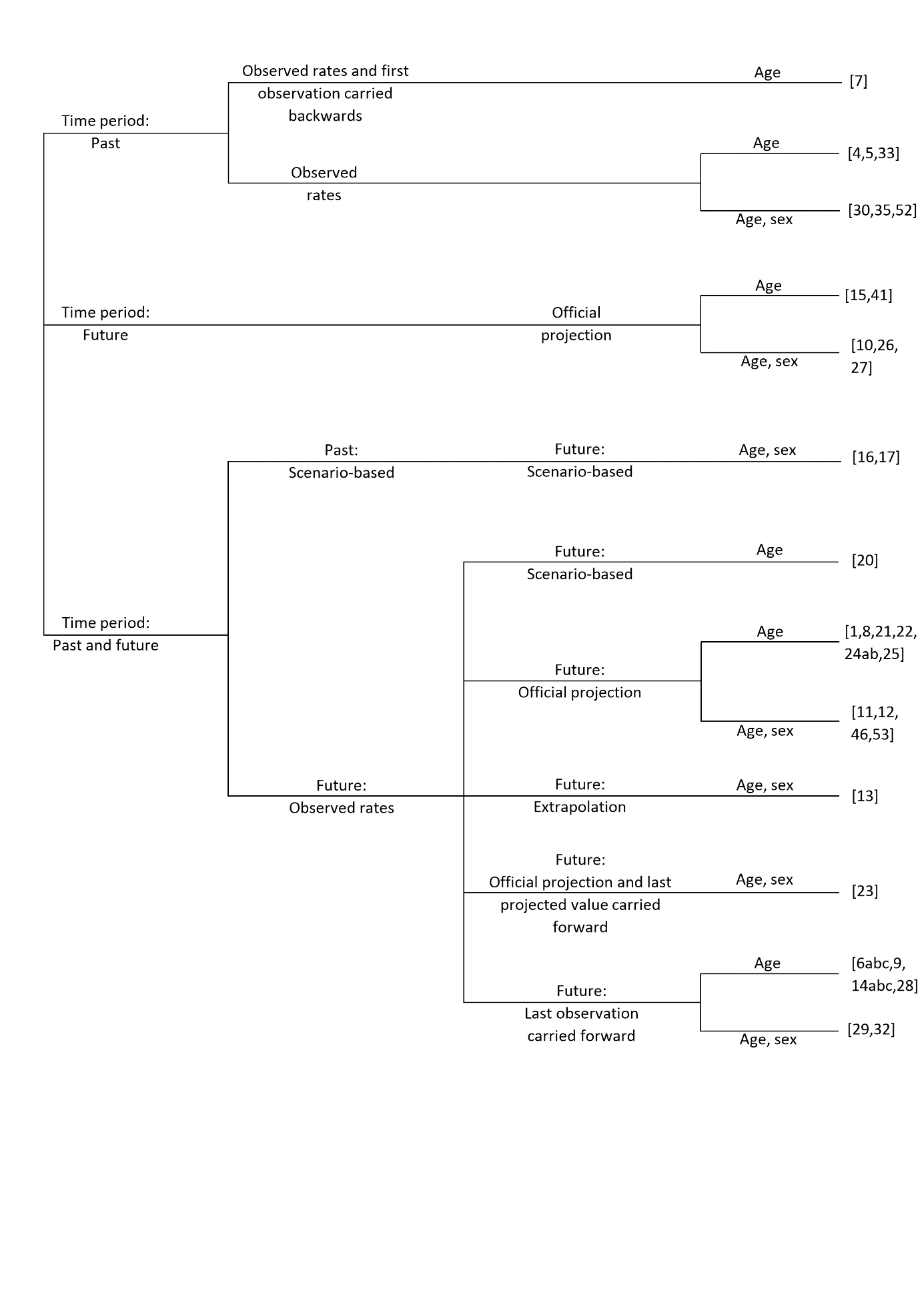


[42]

[10,51]

[41, 56, 62a, 62b, 62c, 16a, 16b, 16c]

[57^S.^,61^S.^,63]

[26,40^S.^,49^S.^,58^S.^]

[44,46]

[9,47^S.^,53^S.^]

[54]

[64, 66]

[25,43a, 43b, 50,52,55,65]

[48,60]

[45]

[59]

*Figure S2: Branching diagram of mortality modelling with article number in brackets. (S.: Spectrum software, 4Flu: 4Flu model).*

| *Table S2: IBMs and CCBMs included in systematic review (NA: not applicable)* | | | | | | | | | | | | | | | | | | | |
| --- | --- | --- | --- | --- | --- | --- | --- | --- | --- | --- | --- | --- | --- | --- | --- | --- | --- | --- | --- |
| **Author** | **Year of  publication** | | **Setting** | | **Time horizon** | | **Demographic characteristics** | | **Demographic data source** | | | **Model type** | **Demographic processes** | | **Demographic rates in  initialisation period** | | | | |
| Mekonnen et al. [40] | 2002 | | Addis Ababa | | 1984-2004 | | Age, sex | | -Central Statistical Authority (Ethiopia)  -UN WPP | | | CCBM | Fertility, mortality, migration | | NA | | | | |
| Gao et al. [41] | 2006 | | China | | 1965-2051 | | Age, sex | | -National Bureau of Statistics (China) -Population Information and Research Center (China) | | | CCBM | Fertility, mortality | | NA | | | | |
| Aparicio et al. [42] | 2009 | | USA (urban population) | | 1850-2009 | | Age | | United States Census Bureau | | | CCBM | Fertility, mortality, migration | | Observation of 1850 (fixed) | | | | |
| Guzzetta et al. [43]a | 2011 | | Arkansas | | 2000-2030 | | Age | | -US Bureau of Census  -US Bureau of Labor Statistics  -US National Center for Education Statistics  -CDC National Vital Statistics | | | CCBM | Fertility, mortality | | NA | | | | |
| McDonald et al. [44] | 2012 | | Netherlands | | 2000-2030 | | Age, sex | | Statistics Netherlands | | | CCBM | Fertility, mortality, migration | | NA | | | | |
| Van Effelterre et al. [45] | 2012 | | Mexico | | 1970-2120 | | Age | | -US Census Bureau -WHO | | | CCBM | Fertility, mortality | | Adjusted to obtain a stable population | | | | |
| McDonald et al. [46] | 2013 | | Netherlands | | 2000-2030 | | Age, sex | | Statistics Netherlands | | | CCBM | Fertility, mortality, migration | | NA | | | | |
| Merler et al. [10] | 2014 | | Italy | | 1901-2009 | | Age | | ISTAT | | | CCBM | Fertility, mortality, migration | | Observation of 1901 (fixed) | | | | |
| Mahy et al. [47] | 2014 | | Nigeria | | 1970-2013 | | Age, sex, state, rural/urban | | UN WPP | | | CCBM | Fertility, mortality, migration | | NA | | | | |
| Knight et al. [48] | 2014 | | 91 low- and middle-income countries | | 2009-2050 | | Age | | UN WPP | | | CCBM | Fertility, mortality | | Observations of 2009 (fixed) | | | | |
| Penazzato et al. [49] | 2014 | | 21 African countries | | 2010-2020 | | Age, sex | | UN WPP | | | CCBM | Fertility, mortality, migration | | NA | | | | |
| Van Effelterre et al. [50] | 2016 | | Thailand | | 1950-2100 | | Age | | -UN WPP -WHO | | | CCBM | Fertility, mortality, internal migration | | Adjusted to obtain a stable population | | | | |
| Costantino et al. [51] | 2017 | | Australia | | 1901-2000 | | Age | | Australian Bureau of Statistics | | | CCBM | Fertility, mortality | | Observation of 1901 (fixed) | | | | |
| Trentini et al. [52] | 2017 | | Australia, Ethiopia, Kenya, Ireland, Italy, South Korea, Singapore, UK, USA | | 1950-2030 | | Age | | UN WPP | | | CCBM | Fertility, mortality, migration | | Observation of 1950 (fixed) | | | | |
| *Table S2: IBMs and CCBMs included in systematic review (NA: not applicable) (continued)* | | | | | | | | | | | | | | | | | | | |
| **Author** | **Year of  publication** | | **Setting** | | **Time horizon** | | **Demographic characteristics** | | **Demographic data source** | | | **Model type** | **Demographic processes** | | **Demographic rates in  initialisation period** | | | | |
| Van Effelterre et al. [25] | 2017 | | Brazil, Mexico | | 1950-2050 | | Age | | -UN WPP -WHO | | | CCBM | Fertility, mortality, internal migration | | Adjusted to obtain a stable population | | | | |
| Mahy et al. [53] | 2017 | | 160 countries | | 1970-2015 | | Age, sex | | UN WPP | | | CCBM | Fertility, mortality, migration | | NA | | | | |
| Williams et al. [54] | 2018 | | Senegal and Gambia as one population | | 1950-2150 | | Age, sex | | UN WPP | | | CCBM | Fertility, mortality | | - Fertility obs. of 1950/55 - Female life table reconstructed to obtain a net reproductive rate of 1 | | | | |
| Jayasundara et al. [55] | 2018 | | Australia | | 1901-2061 | | Age | | Australian Bureau of Statistics | | | CCBM | Fertility, mortality, migration | | Observations of 1901 (fixed) | | | | |
| Mahikul et al. [56] | 2019 | | Thailand | | 1980-2035 | | Age, sex, internal migration group | | -Population and Housing Census -Ministry of Public Health | | | CCBM | Fertility, mortality, migration | | NA | | | | |
| Haacker et al. [57] | 2019 | | Botswana | | 2015-2050 | | Age, sex | | UN WPP | | | CCBM | Fertility, mortality, migration | | NA | | | | |
| Heffernan et al. [26] | 2019 | | 190 countries | | 1950-2100 | | Age, sex | | UN WPP | | | CCBM | Fertility, mortality, migration | | Observation of 1950 (fixed) | | | | |
| Khalifa et al. [58] | 2019 | | 148 countries | | 2010-2050 | | Age, sex | | -UN WPP -National census data | | | CCBM | Fertility, mortality, migration | | NA | | | | |
| Ku et al. [59] | 2019 | | Taiwan | | 2000-2035 | | Age, sex | | Department of Statistics (Taiwan) | | | CCBM | Fertility, mortality, migration | | NA | | | | |
| Trentini et al. [60] | 2019 | | USA, South Korea, Singapore, Australia, Italy, the UK and Ireland | | 2018-2050 | | Age | | UN WPP | | | CCBM | Fertility, mortality | | NA | | | | |
| Yerushalmi et al. [61] | 2019 | | Ghana | | 2015-2045 | | Age, sex | | -UN WPP -DHS | | | CCBM | Fertility, mortality, migration | | NA | | | | |
| Guzzetta et al. [43]b | 2011 | | Arkansas | | 2000-2030 | | Age, household membership, school, workplace | | -US Bureau of Census  -US Bureau of Labor Statistics -US National Center for Education Statistics -CDC National Vital Statistics | | | IBM | Fertility, mortality, household transitions, school/workplace transitions | | NA | | | | |
| Liu et al. [62]a | 2014 | | China | | 1975-2024 | | Age, sex, household membership, school membership, mother link | | Census data, Yearbooks for China | | | IBM | Fertility, mortality, household transitions, school transitions | | NA | | | | |
| *Table S2: IBMs and CCBMs included in systematic review (NA: not applicable) (continued)* | | | | | | | | | | | | | | | | | | | |
| **Author** | **Year of  publication** | | **Setting** | | **Time horizon** | | **Demographic characteristics** | | **Demographic data source** | | | **Model type** | **Demographic processes** | | **Demographic rates in  initialisation period** | | | | |
| Liu et al. [62]b | 2014 | | China | | 1975-2024 | | Age, sex, household membership, schools, mother link | | Census data, Yearbooks for China | | | IBM | Fertility, mortality, household transitions, school transitions | | NA | | | | |
| Liu et al. [62]c | 2014 | | China | | 1975-2024 | | Age, sex, household membership, schools, mother link | | Census data, Yearbooks for China | | | IBM | Fertility, mortality, household transitions, school transitions | | NA | | | | |
| Marziano et al. [16]a | 2015 | | Spain | | 1900-2050 | | Age | | -UN WPP -HMD -INEbase -Eurostat -World Bank | | | IBM | Fertility, mortality, migration | | Observation of 1900 (fixed) | | | | |
| Marziano et al. [16]b | 2015 | | Spain | | 1900-2050 | | Age | | -UN WPP -HMD -INEbase -Eurostat -World Bank | | | IBM | Fertility, mortality, migration | | Observation of 1900 (fixed) | | | | |
| Marziano et al. [16]c | 2015 | | Spain | | 1900-2050 | | Age | | -UN WPP -HMD -INEbase -Eurostat -World Bank | | | IBM | Fertility, mortality, migration | | Observation of 1900 (fixed) | | | | |
| Geard et al. [9] | 2015 | | Australia | | 1910-2010 | | Age, sex, household membership | | -Australian Bureau of Statistics -Survey data | | | IBM | Fertility, mortality, migration, household transitions | | Observations of 1910 (fixed) | | | | |
| Xu et al. [63] | 2017 | | American Samoa | | 2010-2050 | | Age, sex, household membership, residence, household position | | -US Census Bureau -American Samoa Statistical Yearbook 2015 | | | IBM | Fertility, mortality, migration, household transitions | | NA | | | | |
| Campbell et al. [64] | 2017 | | Australia | | 1910-2020 | | Age, sex, household membership | | -Australian Bureau of Statistics -Survey data | | | IBM | Fertility, mortality, household transitions | | Observations of 1910 (fixed) | | | | |
| Melegaro et al. [65] | 2018 | | Italy | | 1900-2100 | | Age | | -HMD -MPIRDR -ISTAT | | | IBM | Fertility, mortality, migration | | Observation of 1900 (fixed) | | | | |
| Smit et al. [66] | 2018 | | Zimbabwe | | 1950-2035 | | Age, sex | | UN WPP | | | IBM | Fertility, mortality | | NA | | | | |
| *Table S3: EPMs included in systematic review (NA: not applicable)* | | | | | | | | | | | | | | | |  |  |  |  |
| **Author** | | **Year of  publication** | | **Setting** | | **Time horizon** | | **Demographic characteristics** | | **Demographic data source** | **Demographic rates in initialisation period** | | | **Population composition** | | |  |  |  |
| Eichner et al. [22] | | 2014 | | Germany | | 1993-2043 | | Age | | DESTATIS | Adjusted to keep age distribution constant | | | Obs. and official projection | | |  |  |  |
| van Lier et al. [67] | | 2015 | | The Netherlands | | 1950-2200 | | Age | | Statistics Netherlands |  | | | -Obs. -Official projection -Official projection carried forward | | |  |  |  |
| Sibley et al. [68] | | 2015 | | 15 countries (Europe, Asia) | | 1950-2030 | | Age, sex | | -UN WPP  -National databases | NA | | | Obs. and official projection | | |  |  |  |
| Schmidt-Ott et al. [23] | | 2016 | | Belgium, Finland, Germany, Great Britain, Italy, Luxembourg, Netherlands and Poland | | 1994-2033 | | Age | | -DESTATIS -Eurostat | Adjusted to keep age distribution constant | | | Obs. and official projection | | |  |  |  |
| Dolk et al. [24] | | 2016 | | Germany | | 1994-2034 | | Age | | DESTATIS | NA | | | Obs. and official projection | | |  |  |  |
| Li et al. [69] | | 2017 | | Six provinces in China | | 1982-2011 | | Age | | National census | NA | | | Obs. (1982, 1990, 2000, 2010) with linear interpolation | | |  |  |  |
| Eichner et al. [70] | | 2017 | | Germany | | 2000-2026 | | Age | | DESTATIS | NA | | | Obs. and official projection (main scenario) | | |  |  |  |
| Hood et al. [71] | | 2017 | | USA | | 2013-2045 | | Age, sex, ethnicity/race | | US Census Bureau | NA | | | Official projection | | |  |  |  |
| Horn et al. [14]a | | 2018 | | Germany | | 1990-2060 | | Age, sex | | DESTATIS | NA | | | Obs. and official projection (medium scenario) | | |  |  |  |
| Horn et al. [14]b | | 2018 | | Germany | | 1990-2060 | | Age, sex | | DESTATIS | NA | | | Obs. and official projection (high migration 2016-2025) | | |  |  |  |
| Arregui et al. [72]a | | 2018 | | 12 countries (Africa, Asia) | | 2000-2050 | | Age | | UN WPP | NA | | | Obs. and official projection | | |  |  |  |
| Arregui et al. [72]b | | 2018 | | 12 countries (Africa, Asia) | | 2015-2050 | | Age | | UN WPP | NA | | | Obs. and synthetic (young, static, ageing) | | |  |  |  |
| Turgeon et al. [73] | | 2018 | | Provinces/territories in Canada | | 1999-2028 | | Age | | Statistics Canada | NA | | | Obs. and official projection (medium growth scenario) | | |  |  |  |
| Schmidt-Ott et al. [21] | | 2019 | | Germany | | 1997-2036 | | Age | | DESTATIS | Adjusted to keep age distribution constant | | | Obs. and official projection | | |  |  |  |
| *Table S3: EPMs included in systematic review (NA: not applicable) (continued)* | | | | | | | | | | | | | | | | | | |  |
| **Author** | | **Year of  publication** | | **Setting** | | **Time horizon** | | **Demographic characteristics** | | **Demographic data source** | **Demographic rates in initialisation period** | | | **Population composition** | | | |  |  |
| Marziano et al. [27] | | 2019 | | Italy | | 2017-2045 | | Age, household membership | | ISTAT | NA | | | -Official projection of age distribution -Household size and age composition of 2017 | | | | | |
| Talbird et al. [74] | | 2020 | | USA (age 50+) | | 2017-2046 | | Age | | US Census Bureau | NA | | | Official projection | | | | | |

| *Table S4: Modelling of fertility (TFR: total fertility rate, ASFR: age-specific fertility rate, CBR: crude birth rate, obs.: observation, NA: not applicable)* | | | | |  |
| --- | --- | --- | --- | --- | --- |
| **Author** | **Measure** | **Past time period** | **Future time period** | **Covariates** | |
| Mekonnen et al. [40] | -TFR -Age distribution (model schedule) -Disease state | Obs. (5-year average) | Last obs. carried forward | Age, sex, disease state | |
| Gao et al. [41] | ASFR | Obs. with interpolation | Last obs. carried forward | Age, sex | |
| Aparicio et al. [42] | Age-specific per capita birth rate | Obs. and linear interpolation | NA | Age | |
| Guzzetta et al. [43]a | CBR | Obs. | Official projection | None | |
| McDonald et al. [44] | ASFR | Obs. for 2000 with a fixed annual increase for ages >27 | Obs. for 2000 with a fixed annual increase for ages >27 | Age, sex | |
| Van Effelterre et al. [45] | Number of births | Obs. | Uncertain. Author no longer has access to information. | None | |
| McDonald et al. [46] | ASFR | Obs. for 2000 with a fixed annual increase for ages >27 | Obs. for 2000 with a fixed annual increase for ages >27 | Age, sex | |
| Merler et al. [10] | CBR | Obs. and linear interpolation | NA | None | |
| Mahy et al. [47] | -TFR -Age distribution -State | Obs. | NA | Age, sex, state | |
| Knight et al. [48] | Number of births | NA | Official projection | None | |
| Penazzato et al. [49] | -TFR -Age distribution (model schedule) -Disease state | Obs. | Official projection | Age, sex, disease state | |
| Van Effelterre et al. [50] | Number of births | Obs. (5-year average) | Official projection (5-year average) | None | |
| Costantino et al. [51] | CBR | Obs. | NA | None | |
| Trentini et al. [52] | CBR | Obs. | Official projection | None | |
| Van Effelterre et al. [25] | Number of births | Obs. with smoothing | Official projection with smoothing | None | |
| Mahy et al. [53] | -TFR -Age distribution (model schedule) | Obs. | NA | Age, sex | |
| Williams et al. [54] | ASFR | Obs. averaged over countries | -Official projections averaged over countries -Last projected value carried forward | Age, sex | |
| Jayasundara et al. [55] | CBR | Obs. | Official projection | None | |
| Mahikul et al. [56] | ASFR | Obs. | Last obs. carried forward | Age, sex | |
| Haacker et al. [57] | TFR Age distribution | NA | Official projection (5-year average) | Age, sex | |
| *Table S4: Modelling of fertility (TFR: total fertility rate, ASFR: age-specific fertility rate, CBR: crude birth rate, obs.: observation, NA: not applicable) (continued)* | | | | | |
| **Author** | **Measure** | **Past time period** | **Future time period** | **Covariates** | |
| Heffernan et al. [26] | ASFR | Obs. (5-year average) | Official projection (5-year average) | Age, sex | |
| Khalifa et al. [58] | TFR Age distribution | Obs. (5-year average) | Official projection (5-year average) | Age, sex | |
| Ku et al. [59] | ASFR | Obs. | Projection with Lee-Carter model | Age, sex | |
| Trentini et al. [60] | CBR | NA | Official projection | None | |
| Yerushalmi et al. [61] | TFR Age distribution | NA | Official projection | Age, sex | |
| Guzzetta et al. [43]b | CBR | Obs. | Official projection | None | |
| Liu et al. [62]a | Age-, parity- and policy-specific fertility rate | Obs. | Last obs. carried forward | Age, sex, parity | |
| Liu et al. [62]b | Age-, parity- and policy-specific fertility rate | Obs. excl. 2nd order births | Last obs. carried forward | Age, sex, parity | |
| Liu et al. [62]c | Age-, parity- and policy-specific fertility rate | Obs. | - Last obs. for 1st order births carried forward - 2nd order birth rate increases to 1st order birth rate | Age, sex, parity | |
| Marziano et al. [16]a | CBR | Obs. | Last obs. carried forward | None | |
| Marziano et al. [16]b | CBR | Obs. | Linear decrease to 0 | None | |
| Marziano et al. [16]c | CBR | Obs. | Linear increase to doubling of last obs. | None | |
| Geard et al. [9] | -Number of births -Age distribution -Birth interval of minimum 270 days | Obs. | NA | Age, sex, parity, birth interval | |
| Xu et al. [63] | ASFR | NA | Official projection | Age, sex | |
| Campbell et al. [64] | -Number of births -Age distribution -Birth interval of minimum 270 days | Obs. | Last obs. carried forward | Age, sex, parity, birth interval | |
| Melegaro et al. [65] | CBR | Obs. | Official projection | None | |
| Smit et al. [66] | ASFR | Obs. (5-year average) | Last obs. carried forward | Age, sex | |

| *Table S5: Modelling of mortality (ASMR: age-specific mortality rate, ASSMR: age-sex-specific mortality rate, obs.: observation, NA: not applicable)* | | | | |
| --- | --- | --- | --- | --- |
| **Author** | **Measure** | **Past time period** | **Future time period** | **Covariates** |
| Mekonnen et al. [40] | -Life expectancy at birth -Model life table -Disease-related mortality | Obs. | Official projection | Age, sex, disease state |
| Gao et al. [41] | ASMR | Obs. with interpolation | Last obs. carried forward | Age |
| Aparicio et al. [42] | -Crude mortality rate -Life table -Disease-related mortality | -First obs. carried backwards (1850-1899) -Function fitted to obs. | NA | Age, disease state |
| Guzzetta et al. [43]a | -ASMR -Disease-related mortality | Obs. | Official projection | Age, disease state |
| McDonald et al. [44] | ASSMR | Obs. 2000 with a fixed annual age-specific decrease | Obs. 2000 with a fixed annual age-specific decrease | Age, sex |
| Van Effelterre et al. [45] | ASMR | Obs. with linear interpolation | Last obs. with annual age-specific adjustment | Age |
| McDonald et al. [46] | ASSMR | Obs. 2000 with a fixed annual age-specific decrease | Obs. 2000 with a fixed annual age-specific decrease | Age, sex |
| Merler et al. [10] | ASMR | Obs. and linear interpolation | NA | Age |
| Mahy et al. [47] | -Life expectancy at birth by sex -Model life table -Disease-related mortality -State | Obs. | NA | Age, sex, state, disease state |
| Knight et al. [48] | ASMR | NA | Official projection | Age, disease state |
| Penazzato et al. [49] | -Life expectancy at birth -Model life table -Disease-related mortality | Obs. | Official projection | Age, sex, disease state |
| Van Effelterre et al. [50] | ASMR | Obs. | Exponentially decreasing function fitted to official projections | Age |
| Costantino et al. [51] | ASMR | Obs. | NA | Age |
| Trentini et al. [52] | ASMR | Obs. with linear interpolation | Official projection with linear interpolation | Age |
| Van Effelterre et al. [25] | ASMR | Obs. | Official projection | Age |
| Mahy et al. [53] | -Life expectancy at birth -Model life table -Disease-related mortality | Obs. | NA | Age, sex, disease state |
| Williams et al. [54] | -ASSMR -Disease-related mortality | Obs. averaged over countries | -Official projections averaged over countries -Last projected value carried forward | Age, sex, disease state |
| *Table S5: Modelling of mortality (ASMR: age-specific mortality rate, ASSMR: age-sex-specific mortality rate, obs.: observation, NA: not applicable) (continued)* | | | | |
| **Author** | **Measure** | **Past time period** | **Future time period** | **Covariates** |
| Jayasundara et al. [55] | ASMR | Obs. (averages over multiple years) with interpolation | Official projection | Age |
| Mahikul et al. [56] | -ASMR -Disease-related mortality | Obs. | Last obs. carried forward | Age, disease state |
| Haacker et al. [57] | -Life expectancy at birth by sex -Model life table -Disease-related mortality | NA | Official projection (5-year average) | Age, sex, disease state |
| Heffernan et al. [26] | -ASSMR -Disease-related mortality -Risk group mortality | Obs. (5-year average) | Official projection (5-year average) | Age, sex, disease state, risk group |
| Khalifa et al. [58] | -Life expectancy at birth by sex -Model life table -Disease-related mortality | Obs. (5-year average) | Official projection (5-year average) | Age, sex, disease state |
| Ku et al. [59] | ASMR | Obs. | Projection with Lee-Carter and Coale-Kisker method | Age, sex |
| Trentini et al. [60] | ASMR | NA | Official projection | Age |
| Yerushalmi et al. [61] | -ASSMR -Disease-related mortality -Regional variation | NA | Official projection | Age, sex, disease state, region |
| Guzzetta et al. [43]b | -ASMR -Disease-related mortality | Obs. | Official projection | Age, disease state |
| Liu et al. [62]a | ASMR | Obs. | Last obs. carried forward | Age |
| Liu et al. [62]b | ASMR | Obs. | Last obs. carried forward | Age |
| Liu et al. [62]c | ASMR | Obs. | Last obs. carried forward | Age |
| Marziano et al. [16]a | ASMR | Obs. | Last obs. carried forward | Age |
| Marziano et al. [16]b | ASMR | Obs. | Last obs. carried forward | Age |
| Marziano et al. [16]c | ASMR | Obs. | Last obs. carried forward | Age |
| Geard et al. [9] | ASSMR | Obs. | NA | Age, sex |
| Xu et al. [63] | -Number of deaths -Life table by sex (fixed) | NA | Official projection | Age, sex |
| Campbell et al. [64] | ASSMR | Obs. | Last obs. carried forward | Age, sex |
| Melegaro et al. [65] | ASMR | Obs. | Official projection | Age |
| *Table S5: Modelling of mortality (ASMR: age-specific mortality rate, ASSMR: age-sex-specific mortality rate, obs.: observation, NA: not applicable) (continued)* | | | | |
| **Author** | **Measure** | **Past time period** | **Future time period** | **Covariates** |
| Smit et al. [66] | -ASSMR -Disease-related mortality | Obs. (5-year average) | Last obs. carried forward | Age, sex, disease state |

| *Table S6: Modelling of migration (obs.: observation, , NA: not applicable)* | | | | |
| --- | --- | --- | --- | --- |
| **Author** | **Measure** | **Past time period** | **Future time period** | **Covariates** |
| Mekonnen et al. [40] | -Net-migration rate -Model age-sex schedule | Obs. of 1984 (fixed) | Last obs. carried forward | Age, sex |
| Aparicio et al. [42] | Net-migration rates (urban population) | Function fitted to obs. | NA | None |
| McDonald et al. [44] | Age-sex-specific net-migration rate | Average obs. 2000-2009 | Average obs. carried forward | Age, sex |
| McDonald et al. [46] | Age-sex-specific net-migration rate | Average obs. 2000-2009 | Average obs. carried forward | Age, sex |
| Merler et al. [10] | -Immigration and emigration rates -Age distribution (fixed) | Obs. | NA | Age |
| Mahy et al. [47] | -Net-migration rate -Model age-sex schedule | Obs. | NA | Age, sex |
| Penazzato et al. [49] | -Net-migration rate -Model age-sex schedule | Obs. | Official projection | Age, sex |
| Van Effelterre et al. [50] | Internal migration rate (urban/rural) | Obs. (5-year average) | Official projection (5-year average) | None |
| Trentini et al. [52] | -Net-migration rate -Age distribution (3 time points only) | Obs. (5-year averages) | Official projection (5-year averages) | Age |
| Van Effelterre et al. [25] | -Internal migration rate (urban/rural) -Net international migration rate (Mexico) | Obs. with smoothing | Official projection with smoothing | None |
| Mahy et al. [53] | -Net-migration rate -Model age-sex schedule | Obs. | NA | Age, sex |
| Jayasundara et al. [55] | -Net-migration rate -Age distribution (average 1976-2015) | Obs. | Official projection | Age |
| Mahikul et al. [56] | -Net international migration rate  -Internal migration rate | Obs. | Last obs. carried forward | Age |
| Haacker et al. [57] | -Net-migration rate -Model age-sex schedule | NA | Official projection (5-year average) | Age, sex |
| *Table S6: Modelling of migration (obs.: observation, , NA: not applicable) (continued)* | | | | |
| **Author** | **Measure** | **Past time period** | **Future time period** | **Covariates** |
| Heffernan et al. [26] | Net-migration rate | Obs. (5-year average) | Official projection (5-year average) | None |
| Khalifa et al. [58] | -Net-migration rate -Model age-sex schedule | Obs. (5-year average) | Official projection (5-year average) | Age, sex |
| Ku et al. [59] | Age-sex-specific net-migration rate | Obs. with residual method | Mean of obs. carried forward | Age, sex |
| Yerushalmi et al. [61] | -Net-migration rate -Model age-sex schedule | NA | Official projection | Age, sex |
| Marziano et al. [16]a | -Net-migration rate -Age distribution (fixed) | Obs. | Last obs. carried forward | Age |
| Marziano et al. [16]b | -Net-migration rate -Age distribution (fixed) | Obs. | Last obs. carried forward | Age |
| Marziano et al. [16]c | -Net-migration rate -Age distribution (fixed) | Obs. | Last obs. carried forward | Age |
| Geard et al. [9] | Net-migration rate | Fixed rate from 1950 | NA | None |
| Xu et al. [63] | -Net-migration rate -Age distribution (fixed) | NA | Official projection | Age |
| Melegaro et al. [65] | Net-migration rate | Obs. | Official projection | Age |

| *Table S7: Households and networks (NA: not applicable)* | | | | |
| --- | --- | --- | --- | --- |
| **Author** | **Household types** | **Transitions** | **Transition rates** | **Other networks** |
| Guzzetta et al. [43]b | Single person household, married couple with/without child(ren), single(s) with/without child(ren), other households | Marriage, divorce, leaving current household to create a new | Dynamic | Schools, workplaces membership, spatial location of households, schools and workplaces |
| Liu et al. [62]a | Union with/without child(ren), single adult with/without child(ren), non-family related individuals with/without nuclear family | Union formation, leaving parental household | Fixed over time and equal across eligible ages | School links |
| Liu et al. [62]b | Union with/without child(ren), single adult with/without child(ren), non-family related individuals with/without nuclear family | Union formation, leaving parental household | Fixed over time and equal across eligible ages | School links |
| Liu et al. [62]c | Union with/without child(ren), single adult with/without child(ren), non-family related individuals with/without nuclear family | Union formation, leaving parental household | Fixed over time and equal across eligible ages | School links |
| *Table S7: Households and networks (NA: not applicable) (continued)* | | | | |
| **Author** | **Household types** | **Transitions** | **Transition rates** | **Other networks** |
| Geard et al. [9] | Union with/without child(ren), single parent with child(ren), single | Union formation and dissolution, child leaving parental household | Fixed and equal across eligible ages | NA |
| Xu et al. [63] | Couple with/without child(ren), single with/without child(ren), non-related adult(s), non-related adult(s) living with nuclear family, multi-generational household, institutions | Union formation and dissolution, senior individuals moving to household of adult child, single person household to institution | Fixed and equal across ages | Household are assigned a location while ensuring plausible living space per capita |
| Campbell et al. [64] | Union with/without child(ren), single parent with child(ren), single | Union formation and dissolution, child leaving parental household | Time-dependent but equal across eligible ages | NA |
| Marziano et al. [27] | Couple with/without child(ren), single adult with child(ren) Household size 1-7 | New households are generated each year according to the household size and age composition of 2017. | NA | NA |

| *Table S8: Sensitivity analyses* | | | |
| --- | --- | --- | --- |
| **Author** | **Demographic processes** | **Time period** | **Method** |
| Mekonnen et al. [40] | Fertility | 2000-2024 | Scenario-based (low/high) |
| Van Effelterre et al. [50] | Fertility and internal migration | 1950-2100 | Interpolation between 5-year averages |
| Williams et al. [54] | Fertility | 2015-2100 | Scenario-based |
| Ku et al. [59] | Fertility, mortality, migration | 2018-2035 | Prediction intervals |
| Marziano et al. [16]a | Fertility, mortality, migration | 2010-2050 | Scenario-based (UN medium variant) |
| Marziano et al. [16]b | Fertility, mortality, migration | 2010-2050 | Scenario-based (UN medium variant) |
| Marziano et al. [16]c | Fertility, mortality, migration | 2010-2050 | Scenario-based (UN medium variant) |
| Melegaro et al. [65] | Fertility | 2016-2100 | Scenario-based |
| Arregui et al. [72]a | Age distribution | 2015-2050 | Confidence interval, alternative age distributions (young/old population) |

| *Table S9: Infectious disease modelling (CEA: cost-effectiveness analysis, NA: not applicable)* | | | | |
| --- | --- | --- | --- | --- |
| **Author** | **Disease** | **Vaccination** | **Social mixing** | **CEA** |
| Mekonnen et al. [40] | HIV/AIDS | No | No | No |
| Gao et al. [41] | Rubella | Yes | Proportionate mixing | NA |
| Aparicio et al. [42] | Tuberculosis | NA | NA | NA |
| Guzzetta et al. [43]a | Tuberculosis | NA | Homogeneous mixing | NA |
| McDonald et al. [44] | Hepatitis B, influenza | Yes | NA | NA |
| Van Effelterre et al. [45] | Hepatitis A | Yes | WAIFW matrix | NA |
| McDonald et al. [46] | Hepatitis A | Yes | NA | NA |
| Merler et al. [10] | Measles | Yes | Homogeneous mixing and sensitivity analysis (POLYMOD) | NA |
| Mahy et al. [47] | HIV/AIDS | No | No | No |
| Knight et al. [48] | Tuberculosis | Yes | No | Yes |
| Penazzato et al. [49] | HIV/AIDS | No | No | No |
| Van Effelterre et al. [50] | Hepatitis A | NA | Homogeneous mixing | NA |
| Costantino et al. [51] | Varicella zoster virus | NA | POLYMOD | NA |
| Trentini et al. [52] | Measles | Yes | Homogeneous mixing | NA |
| Van Effelterre et al. [25] | Hepatitis A | NA | Homogeneous mixing | NA |
| Mahy et al. [53] | HIV/AIDS | No | No | No |
| Williams et al. [54] | Hepatitis B | Yes | WAIFW matrix | NA |
| Jayasundara et al. [55] | Hepatitis A | Yes | POLYMOD | NA |
| Mahikul et al. [56] | Melioidosis | NA | NA | NA |
| Haacker et al. [57] | HIV and non-communicable diseases | NA | NA | NA |
| Heffernan et al. [26] | Hepatitis C | NA | NA | NA |
| Khalifa et al. [58] | HIV | NA | NA | NA |
| Ku et al. [59] | Tuberculosis | NA | NA | NA |
| Trentini et al. [60] | Measles | Yes | Homogeneous mixing | NA |
| Yerushalmi et al. [61] | Malaria | Yes | NA | Yes |
| *Table S9: Infectious disease modelling (CEA: cost-effectiveness analysis, NA: not applicable)* | | | | |
| **Author** | **Disease** | **Vaccination** | **Social mixing** | **CEA** |
| Guzzetta et al. [43]b | Tuberculosis | No | Heterogeneous mixing | NA |
| Liu et al. [62]a | Influenza | No | Household contacts, school contacts and social contacts. | NA |
| Liu et al. [62]b | Influenza | No | Household contacts, school contacts and social contacts. | NA |
| Liu et al. [62]c | Influenza | No | Household contacts, school contacts and social contacts. | NA |
| Marziano et al. [16]a | Varicella zoster virus | Yes | Synthetic social contact matrices | No |
| Marziano et al. [16]b | Varicella zoster virus | Yes | Synthetic social contact matrices | No |
| Marziano et al. [16]c | Varicella zoster virus | Yes | Synthetic social contact matrices | No |
| Geard et al. [9] | "measles-like" illness | Yes | Dynamic contact matrix based on POLYMOD | NA |
| Xu et al. [63] | Lymphatic filariasis (not modelled) | No | NA | NA |
| Campbell et al. [64] | Pertussis | Yes | Dynamic contact matrix based on POLYMOD | NA |
| Melegaro et al. [65] | Varicella and herpes zoster | Yes | Synthetic social contact matrices | Yes |
| Smit et al. [66] | HIV, non-communicable diseases | No | NA | NA |
| Eichner et al. [22] | Influenza | Yes | Dynamic contact matrix based on POLYMOD | NA |
| van Lier et al. [67] | Varicella and herpes zoster | Yes | Yes (POLYMOD) | Yes |
| Sibley et al. [68] | Hepatitis C | No | NA | No |
| Schmidt-Ott et al. [23] | Influenza | Yes | Dynamic contact matrix based on POLYMOD | NA |
| Dolk et al. [24] | Influenza | Yes | Dynamic contact matrix based on POLYMOD | Yes |
| Li et al. [69] | Measles | Yes | No | No |
| Eichner et al. [70] | Influenza | Yes | Yes (POLYMOD) | No |
| Hood et al. [71] | HIV | No | No | No |
| Horn et al. [14]a | Varicella zoster virus | Yes | POLYMOD | NA |
| Horn et al. [14]b | Varicella zoster virus | Yes | POLYMOD | NA |
| Arregui et al. [72]a | Tuberculosis | No | Heterogeneous mixing | No |
| Arregui et al. [72]b | Tuberculosis | No | Heterogeneous mixing | No |
| Turgeon et al. [73] | Salmonella | No | No | No |
| *Table S9: Infectious disease modelling (CEA: cost-effectiveness analysis, NA: not applicable)* | | | | |
| **Author** | **Disease** | **Vaccination** | **Social mixing** | **CEA** |
| Schmidt-Ott et al. [21] | Influenza | Yes | Dynamic contact matrix based on POLYMOD | NA |
| Marziano et al. [27] | Measles | Yes | POLYMOD | NA |
| Talbird et al. [74] | Influenza, pertussis, herpes zoster, and pneumococcal disease | Yes | No | Yes |
